# Supplementary material for: Defining the Role of the miR-145—KLF4—αSMA Axis in Mitral Valvular Interstitial Cell Activation in Myxomatous Mitral Valve Prolapse Using the Canine Model
Source: Int J Mol Sci. 2024 Jan 25;25(3):1468. doi: 10.3390/ijms25031468 (PMC10855421; doi:10.3390/ijms25031468)

Supplemental Data

Supplemental Figure S1. aSMA expression level by PCR analyzed by age (left) and weight (right) groups did not show any significant differences as analyzed by one-way ANOVA. The Ct numbers are normalized to the housekeeping genes (HPRT and RPS19). n = 9 (<5 year old), 7 (6-10 year old), 20 (11-15 year old), 2 (> 15 year old), 15 (< 10 kg), 5 (11-20 kg), 6 (21-30 kg), 3 (> 30 kg).

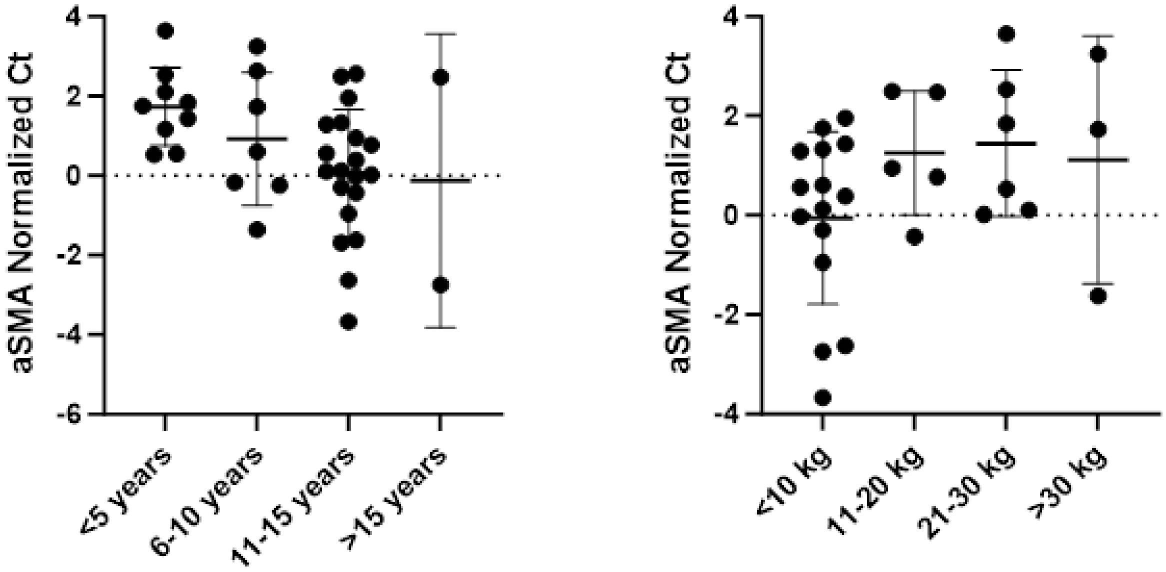

Supplement: Supplementary file 1 [file ijms-25-01468-s001.zip › ijms-2826939-supplementary.pdf]
